# Mixed Infections of* Helicobacter pylori* Isolated from Patients with Gastrointestinal Diseases in Taiwan

**DOI:** 10.1155/2016/7521913

**Published:** 2016-09-22

**Authors:** Chih-Ho Lai, Ju-Chun Huang, Chuan Chiang-Ni, Ju-Pi Li, Lii-Tzu Wu, Hua-Shan Wu, Yu-Chen Sun, Mei-Ling Lin, Ju-Fang Lee, Hwai-Jeng Lin

**Affiliations:** ^1^Graduate Institute of Biomedical Sciences, Department of Microbiology and Immunology, College of Medicine, Chang Gung University, Taoyuan, Taiwan; ^2^Molecular Infectious Disease Research Center, Department of Pediatrics, Chang Gung Children's Hospital and Chang Gung Memorial Hospital, Taoyuan, Taiwan; ^3^Graduate Institute of Basic Medical Science, School of Medicine, China Medical University, Taichung, Taiwan; ^4^Department of Medical Research and Department of Laboratory Medicine, China Medical University and Hospital, Taichung, Taiwan; ^5^Department of Nursing, Asia University, Taichung, Taiwan; ^6^Rheumatology Research Center, China Medical University Hospital, Taichung, Taiwan; ^7^Department of Laboratory Medicine, Chang Gung Memorial Hospital, Taoyuan, Taiwan; ^8^Division of Gastroenterology and Hepatology, Department of Internal Medicine, School of Medicine, College of Medicine, Taipei Medical University, Taipei, Taiwan; ^9^Division of Gastroenterology and Hepatology, Department of Internal Medicine, Shuang-Ho Hospital, New Taipei, Taiwan

## Abstract

*Background*. Persistent* Helicobacter pylori* infection may induce several upper gastrointestinal diseases. Two major virulence factors of* H. pylori*, vacuolating cytotoxin A (VacA) and cytotoxin-associated gene A (CagA), are thought to be associated with the severity of disease progression. The distribution of* vacA* and* cag*-pathogenicity island (*cag*-PAI) alleles varies in* H. pylori* isolated from patients in different geographic regions.* Aim*. To assess the association between mixed infection of* H. pylori* clinical isolates from Taiwanese patients and the severity of gastrointestinal diseases.* Methods*. A total of 70 patients were enrolled in this study. Six distinct and well-separated colonies were isolated from each patient and 420 colonies were analyzed to determine the genotypes of virulence genes.* Results*. The prevalence of mixed infections of all* H. pylori*-infected patients was 28.6% (20/70). The rate of mixed infections in patients with duodenal ulcer (47.6%) was much higher than that with other gastrointestinal diseases (*P* < 0.05).* Conclusions*.* H. pylori* mixed infections show high genetic diversity that may enhance bacterial adaptation to the hostile environment of the stomach and contribute to disease development.

## 1. Introduction

*Helicobacter pylori *is a gram-negative, spiral shaped microaerophilic bacterium that colonizes the human gastric mucosa throughout life [1]. Persistent* H. pylori* infection is associated with several gastrointestinal disorders, including chronic gastritis, peptic ulcer, lymphoid tissue lymphoma, and gastric adenocarcinoma [2]. It has been reported that* H. pylori *may select a particular niche on the mucosa where the bacteria can evade host immune responses by utilizing delicate strategies to manipulate immune cells as well as protect against antibiotic attack, leading to the progression of gastrointestinal diseases [3, 4].

Several virulence factors involving* H. pylori*-induced pathogenesis and the underlying mechanisms have led to different clinical sequelae [5–7]. Vacuolating cytotoxin (VacA), one of the major virulence factors secreted from* H. pylori*, has been detected in bacterial culture supernatants [8]. Upon* H. pylori* colonization on cells, bacterial surface-contacted VacA is secreted directly from bacteria, followed by the intoxication of cells by vacuolation [9]. Previous studies reported that* vacA* was diversified among clinical* H. pylori* isolates, particularly in the region encoding the signal sequence (type s1 or s2) and the mid-region (type m1 or m2) [10]. Additionally, the distribution of* vacA* alleles varies among different geographic regions [11–14].

Another virulence factor of* H. pylori* is the* cag*-pathogenicity island- (*cag*-PAI-) encoded type four secretion system, which mediates the translocation of cytotoxin-associated gene A (CagA) into host cells [15, 16]. Once translocated into cells, CagA is phosphorylated at one or more tyrosine phosphorylation motifs to induce cell pathogenesis [17]. Diversity within* cag*-PAI is found among people from Eastern and Western parts of the world [18]. Nearly all East Asian isolates carry* cag*-PAI, and one-half to two-thirds of the isolates from Western countries carry* cag*-PAI [19, 20]. Of note,* cagA*,* cagE*, and* cagT* were found to be present in 100% of the domestic strains isolated from patients in Taiwan [21]. These findings indicate that* H. pylori* isolates possess unusually high genetic heterogeneity and are diverse in different geographic regions.

*H. pylori* mixed infections have been found to involve more than one allele of either the s-region or m-region of* vac*A [22–24]. The rates of mixed infections may differ in* cag*-PAI of* H. pylori* isolated from the corpus and antrum [22] or there may be discrepancies in the antimicrobial susceptibility tests [25]. The rates of mixed infections vary from 0% to 85% in different populations worldwide [14, 22, 26–28]. However, the prevalence of* H. pylori* mixed infections isolated from patients in Taiwan remains unknown. In this study, we characterized six isolates from each patient using genotyping analysis. The association between mixed infections in* H. pylori* clinical isolates from Taiwanese patients and disease severity was assessed.

## 2. Materials and Methods

### 2.1. Patient Selection

From January 2011 to December 2014, a total of 70 patients with* H. pylori* infection were selected and diagnosed with upper gastrointestinal problems. Patients were excluded if they presented with any of the following: unwillingness to give written informed consent; bleeding tendency; and usage of H_2_-receptor antagonists or proton pump inhibitors within two weeks of enrollment [29].* H. pylori *status was assessed by [^13^C] urea breath test and bacterial culture was performed on biopsies before therapy [30]. Among the enrolled patients, there were 9 patients with chronic gastritis, 21 with duodenal ulcer, 22 with gastric ulcer, and 18 with gastric carcinoma. The severity of gastroenterological disorders was evaluated using endoscopic examination and confirmed by histology as previously described [29]. All the patients had completed a self-administered questionnaire prior to being enrolled in the study. This study was approved by the Clinical Research Committee of Taipei Medical University, Taipei, Taiwan.

### 2.2. *H. pylori* Isolates and Bacterial Culture

Two biopsied specimens of each patient were taken: one specimen from antrum (lesser curvature side) and another from low body (greater curvature side).* H. pylori* isolates were cultured from the biopsies specimen and identified by biochemical reactions [24].* H. pylori* were diagnosed with positive reaction in catalase, urease, and oxidase tests. The bacterial isolates were routinely cultured on Brucella agar plates (Becton Dickinson, Franklin Lakes, NJ) with appropriate antimicrobial agents as described previously [31].

### 2.3. Preparation of Genomic DNA and Polymerase Chain Reaction

After obtaining positive cultures from the biopsies, 6 isolated colonies from a single culture plate were examined for the genotypes using polymerase chain reaction (PCR) approach as described previously [29, 30, 32]. Briefly, the genomic DNA was extracted from the colonies by the sterile micropestle in guanidinium isothiocyanate, and the prepared DNA was dissolved in 10 mM Tris-HCl (pH 8.3). Two microliters of the eluted DNA was subjected to each PCR reaction. Twelve paired primers (Table 1) were then used to amplify specific DNA fragments. The PCR was performed under the following condition: 30 cycles at 94°C for 1 min, 50.9–63°C for 2 min, 72°C for 1 min, and final extension at 72°C for 5 min. Mixed infection was defined as distinct expression of* cagA*,* cagE*,* cagT*,* cagM*, and* vacA *s- or m-regions among the 6 isolates isolated from one host.

### 2.4. Statistical Analysis

The relationship of between-group comparisons was performed using the Chi-square test with Fisher's exact test. A *P* value of less than 0.05 was considered significant.

## 3. Results

From January 2011 to December 2014, 70 patients diagnosed with upper gastrointestinal diseases and* H. pylori*-positive status (9 with chronic gastritis, 21 with duodenal ulcer, 22 with gastric ulcer, and 18 with gastric carcinoma) were enrolled in this study. From each patient, the biopsies were taken from the antrum and body of stomach. We obtained 6 colonies from pooled isolates in one culture plate, for a total of 420 isolates, to study the* H. pylori* genes.* cag*-PAI status and the s-region or m-region of* vac*A in* H. pylori *were assessed by PCR using unique primers. Mixed infection was defined as distinct expression of* cagA*,* cagE*,* cagT*,* cagM*, and* vacA *s- or m-regions among the 6 isolates in one host. The PCR distribution of positive/negative results for individual genes was shown in Table 2. All the isolates from single infection (*n* = 300) and mixed infection (*n* = 120) were positive for* cagA*. There were no major differences of the gene distributions between the two groups.

We then analyzed the association between mixed infections in* H. pylori* clinical isolates from patients and disease severity. As shown in Table 3, a total of 20 patients with* H. pylori* mixed infections, two patients (22.2%) with chronic gastritis, 10 patients (47.6%) with duodenal ulcer, 4 patients (18.2%) with gastric ulcer, and 4 patients (22.2%) with gastric carcinoma, had mixed infections. Additionally, patients with duodenal ulcer showed a higher prevalence of* H. pylori* mixed infection compared to that in other gastrointestinal diseases (*P* < 0.05). In all studied subjects, mixed infections of* H. pylori* strains were found in 20 (28.6%) patients.

## 4. Discussion

*H. pylori* mixed infections were defined as having more than one allele of either the s-region or m-region of* vac*A or both* ice*A1 and* ice*A2 genotypes [22–24]. The rates of mixed infections were varied from 0% to 85% [14, 22, 26–28]. However, most studies used pooled cultures or biopsies for PCR, which may have yielded misleading results. Additionally, the sampling methods, including bacterial cultures from the sites of biopsies and analysis of the antimicrobial-resistant fractions or unselected strains, might have been attributed to the discrepant results [33]. In this study, we used 6 distinct colonies from each culture plate for genotyping of the* cagA*,* cagE*,* cagT*,* cagM*, and* vacA *s- or m-regions. We evaluated a large number of colonies (*n* = 420) isolated from 70 patients. Moreover, 11 pairs of primers, including those for the genes* cag*-PAI and* vacA *s- or m-regions, were used. Therefore, we accurately and sensitively analyzed* H. pylori* mixed infections.

Mixed genotypes were found in 24% of patients who were Chinese residents of Hong Kong [33]. Another report indicated that the prevalence of mixed infections was 23.3% of all* H. pylori*-infected samples isolated from patients who lived in southern Taiwan [28]. In this study, we found that the prevalence of* H. pylori* mixed infections was 28.6% (20/70) in patients who were residents of northern Taiwan. This discrepancy in the prevalence of mixed infections in Chinese populations may be explained by the different analysis strategies and the fact that enrolled subjects were from different geographic regions [34, 35].

A high percentage of subjects (77%) carrying a mix of metronidazole-susceptible/resistant strains have been reported [33]. Mixed infections with metronidazole-resistant strains may not be eradicated by metronidazole-based therapy [36–38]. Additionally, patients with mixed infections in the corpus showed a significantly higher rate of intestinal metaplasia in the antrum [28]. Our study showed that patients with duodenal ulcer have higher rates of mixed infections than of chronic gastritis. These findings support those of previous studies indicating that mixed infections facilitate interstrain gene transfer and genetic diversity, enhancing* H. pylori* survival in the harsh environment of the stomach where disease progression occurs [39, 40].

In conclusion, our study reported that the prevalence of* H. pylori* mixed infections was high in residents living in northern Taiwan and that the rates differed from those in other Chinese populations from other geographic regions. The mixed infections in* H. pylori* with high genetic diversity may promote bacterial adaptation to the stomach and contribute to disease development.

## Figures and Tables

**Table 1:** PCR primers used in this study.

Gene	Primer	Nucleotide sequence (5′-3′)	Length of PCR product
*cag A*	cagA-F	GATAACAGGCAAGCTTTTGAGG	349
	cagA-R	CTGCAAAAGATTGTTTGGCAGA	

*cag E*	cagE-F	GTTACATCAAAAATAAAAGGAAGCG	735
	cagE-R	CAATAATTTTGAAGAGTTTCAAAGC	

*cag T*	cagT-F	TCTAAAAAGATTACGCTCATAGGCG	490
	cagT-R	CTTTGGCTTGCATGTTCAAGTTGCC	

*cag M*	cagM-F	ACAAATACAAAAAAGAAAAAGAGGC	587
	cagM-R	ATTTTTCAACAAGTTAGAAAAAGCC	

*s1 and s2*	VA1-F	ATGGAAATACAACAAACACACC	259
	VA1-R	CTGCTTGAATGCGCCAAACTTTATC	286

*s1a*	SS1-F	GTCAGCATCACACCGCAAC	190

*s1b*	SS3-F	AGCGCCATACCGCAAGAG	187

*s1c*	S1C-F	CTTGCTTTAGTTGGGTTA	213

*m1*	VA3-F	GGTCAAAATGCGGTCATGG	290
	VA3-R	CCATTGGTACCTGTAGAAAC	

*m1T*	m1T-F	GGTCAAAATGCGGTCATGG	290
	m1T-R	CTCTTAGTGCCTAAAGAAACA	

*m2*	VA4-F	GGAGCCCCAGGAAACATTG	352
	VA4-R	CATAACTAGCGCCTTGCAC	

**Table 2:** PCR analysis for *cag*-PAI status and the s-region or m-region of *vac*A in single and mixed infections of *H. pylori*.

Gene	Single infection, *n* = 300 (%)	Mixed infection, *n* = 120 (%)
*cag A*	300 (100.0)	120 (100.0)
*cag E*	295 (98.3)	110 (91.7)
*cag T*	296 (98.7)	116 (96.7)
*cag M*	300 (100.0)	114 (95.0)
*vacA s1a*	300 (100.0)	117 (97.5)
*vacA s1c*	273 (91.0)	100 (83.3)
*vacA m1T*	110 (36.7)	53 (44.2)
*vacA m2*	214 (71.3)	99 (82.5)

**Table 3:** Prevalence of mixed *H. pylori* infections in patients with chronic gastritis, gastric ulcer, duodenal ulcer, and gastric carcinoma.

Diagnosis (*n* = 70)	Number of *H. pylori* mixed infections
Chronic gastritis (*n* = 9)	2 (22.2%)
Gastric ulcer (*n* = 22)	4 (18.2%)
Duodenal ulcer (*n* = 21)	10 (47.6%)^*∗*^
Gastric carcinoma (*n* = 18)	4 (22.2%)

^*∗*^ *P* < 0.05, duodenal ulcer versus chronic gastritis, gastric ulcer, and gastric carcinoma.
